# Acute Effect of Caffeine on the Synthesis of Pro-Inflammatory Cytokines in the Hypothalamus and Choroid Plexus during Endotoxin-Induced Inflammation in a Female Sheep Model

**DOI:** 10.3390/ijms222413237

**Published:** 2021-12-08

**Authors:** Aleksandra Szczepkowska, Maciej Wójcik, Dorota Tomaszewska-Zaremba, Hanna Antushevich, Agata Krawczyńska, Wiktoria Wiechetek, Janina Skipor, Andrzej Przemysław Herman

**Affiliations:** 1 Institute of Animal Reproduction and Food Research, Polish Academy of Sciences, 10-748 Olsztyn, Poland; a.szczepkowska@pan.olsztyn.pl; 2 The Kielanowski Institute of Animal Physiology and Nutrition, Polish Academy of Sciences, 05-110 Jabłonna, Poland; m.wojcik@ifzz.pl (M.W.); d.tomaszewska@ifzz.pl (D.T.-Z.); a.antuszewicz@ifzz.pl (H.A.); a.krawczynska@ifzz.pl (A.K.); w.wiechetek@ifzz.pl (W.W.); 3 Department of Ichthyology and Biotechnology in Aquaculture, Institute of Animal Sciences, University of Life Sciences, 02-786 Warsaw, Poland

**Keywords:** brain, LPS, IL-1β, IL-6, TNFα, *IL1R1*, *IL1R2*, *IL6R*, *IL6ST*, *TNFRSF1A*, *TNFRSF1B*

## Abstract

This study was designed to determine the effect of acute caffeine (CAF) administration, which exerts a broad spectrum of anti-inflammatory activity, on the synthesis of pro-inflammatory cytokines and their receptors in the hypothalamus and choroid plexus (ChP) during acute inflammation caused by the injection of bacterial endotoxin—lipopolysaccharide (LPS). The experiment was performed on 24 female sheep randomly divided into four groups: control; LPS treated (iv.; 400 ng/kg of body mass (bm.)); CAF treated (iv.; 30 mg/kg of bm.); and LPS and CAF treated. The animals were euthanized 3 h after the treatment. It was found that acute administration of CAF suppressed the synthesis of interleukin (IL-1β) and tumor necrosis factor (TNF)α, but did not influence IL-6, in the hypothalamus during LPS-induced inflammation. The injection of CAF reduced the LPS-induced expression of TNF mRNA in the ChP. CAF lowered the gene expression of IL-6 cytokine family signal transducer (*IL6ST*) and TNF receptor superfamily member 1A (*TNFRSF1*) in the hypothalamus and IL-1 type II receptor (*IL1R2*) in the ChP. Our study on the sheep model suggests that CAF may attenuate the inflammatory response at the hypothalamic level and partly influence the inflammatory signal generated by the ChP cells. This suggests the potential of CAF to suppress neuroinflammatory processes induced by peripheral immune/inflammatory challenges.

## 1. Introduction

The inflammation caused by a bacterial or viral infection often influences the activity of neurons located in different hypothalamic nuclei that centrally regulate thermogenesis, food intake, reproduction, and circadian rhythms of rest-activity and sleep [1,2,3,4]. Bacterial endotoxins have been observed to disturb the mechanism regulating reproductive processes in cows [5], sheep [6], pigs [7], rats [8], and non-human primates [9]. Moreover, it has been found that inflammation induced by pathogenic bacteria may trigger premature uterine contractions causing pre-term delivery [10]. Actually, immune stress is thought to alter the reproductive process mainly at the level of the brain [11]. The pivotal role in the transmission of inflammatory signals from the periphery into the brain parenchyma play pro-inflammatory cytokines, particularly interleukin (IL)-1β, IL-6, and tumor necrosis factor (TNF)α, which concentration raises in the peripheral blood, cerebrospinal fluid (CSF) and brain parenchyma in response to lipopolysaccharide (LPS)-induced inflammation [12,13,14,15]. Studies on rats and sheep models indicated that both systemic LPS treatment and central administration of IL-1β, and TNFα into the region of the hypothalamus suppress the secretion of the gonadotropin-releasing hormone leading to downstream inhibition of the hypothalamic-pituitary-gonadal axis (HPG axis), the main neuroendocrine axis regulating reproduction [13,16,17,18]. On the other hand, pharmacological inhibition of the synthesis of pro-inflammatory cytokines in the hypothalamus during endotoxin-induced immune challenge has been shown to reduce the inhibitory effect of inflammation on the activity of the HPG axis in sheep during the follicular phase [19].

Although the area of the brain is protected against an uncontrolled influx of peripheral molecules by the blood-brain barrier (BBB) located in the endothelium of brain microvessels and by the blood-CSF-barrier (BCSFB) in the epithelium of the choroid plexus (ChP), IL-1β, TNFα, and IL-6 cross brain barriers through saturable transport mechanisms [20,21]. Moreover, pro-inflammatory cytokines are also synthesized in the ChP, brain microvessels, and parenchyma in response to peripheral LPS administration [22,23,24]. The ChP serves as a place for elaboration of signal molecules that communicate peripheral immune status to the brain, and as a gateway for immune cells trafficking into the CSF [25,26].

The growing evidence suggests that caffeine (CAF), a plant alkaloid structurally related to adenosine, has an anti-inflammatory action [27,28,29]. Long-term CAF administration decreases the expression of IL-1β, IL-6, and TNF-α in monocytes and macrophages, indicating the possibility of a chronic decrease of local inflammation [30,31]. The ability of CAF to attenuate pro-inflammatory cytokines expression may result from the fact that it binds with adenosine receptors (ADORs), which belong to the P1 family of purinergic G protein-coupled receptors including A1, A2A, A2B, and A3 receptors [32]. CAF demonstrates the highest affinity for A1 and A2A, and the lowest affinity for A2B and A3 [33]. The expression of ADORs has been demonstrated in the brain [34], including ChP in rodents [35,36], pig [35], and human [37,38].

Therefore, based on the assumptions that CAF receptors are widespread in the brain, and CAF may influence pro-inflammatory cytokines synthesis in the immune cells, we hypothesized that CAF decreases the immune response to LPS-induced acute inflammation in the hypothalamus and ChP. With the use of the female sheep model, which exhibits similar sensitivity to LPS [39] and has a diurnal activity pattern as humans [40], we evaluated the effect of acute administration of CAF on the expression of pro-inflammatory cytokines (IL-1β, IL-6, and TNF-α) and their receptors (IL-1 receptor type I—IL1R1 and type II—IL1R2, IL-6 receptor—IL6R and signal-transducing component—IL6ST, TNFα receptor type I—TNFRSF1A and type II—TNFRSF1B) in the hypothalamus and ChP during an immune/inflammatory challenge induced in the follicular phase of the estrous cycle.

## 2. Results

### 2.1. The Effect of Caffeine on Gene Expression and Concentration of Pro-Inflammatory Cytokines in the Hypothalamus under Basal and LPS-Challenge Conditions

**Figure 1:**
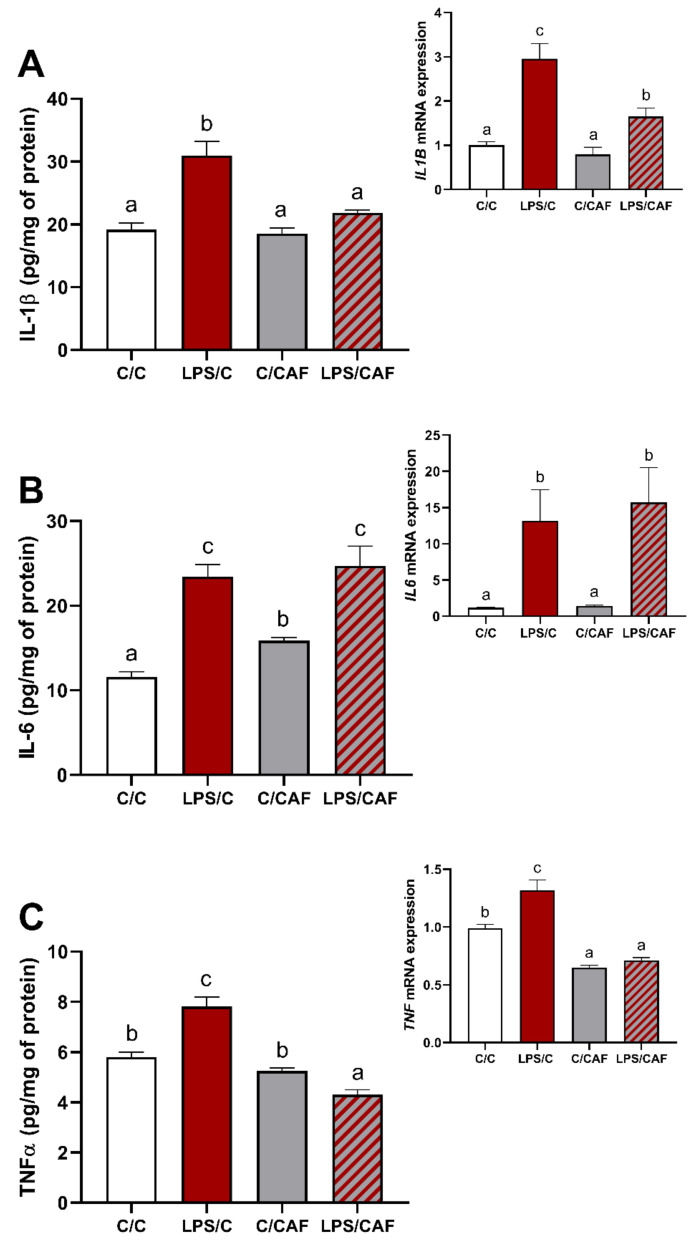
Mean (±SEM) concentration and gene expression (insert) of pro-inflammatory cytokines: interleukin 1beta (**A**), interleukin 6 (**B**) and tumor necrosis factor-alpha (**C**) in the ovine hypothalamus in saline- (C/C, 0.9% NaCl iv., white bars), lipopolysaccharide- (LPS/C, 400 ng/kg of body mass (bm.), iv., red bars), caffeine- (C/CAF, 30 mg/kg of bm., iv., grey bars) and LPS/CAF- (red-grey hatched bars) treated female sheep. Different letters indicate significant differences at *p* < 0.05, according to one-way ANOVA followed by Fisher’s post hoc test comparing groups with each other.

All LPS-treated female sheep responded with a significant increase (*p* < 0.05) of body temperature and blood plasma cortisol concentration (Supplementary Figure S1). At the hypothalamus level, the single injection of LPS affected (*p* < 0.05) the expression of all examined pro-inflammatory cytokines: IL-1β, IL-6, and TNFα, as indicated by higher (*p* < 0.05) mRNA expression together with an increase (*p* < 0.05) of cytokines concentration in the LPS/C group compared to the control (Figure 1A–C with inserts). In turn, the single injection of CAF had no effect on IL-1β expression on both mRNA and protein levels (Figure 1A with insert), as well as on *IL6* mRNA expression (Figure 1B insert) and TNFα protein level (Figure 1C), but increased (*p* < 0.05) the protein level of IL-6, and decreased (*p* < 0.05) the mRNA expression of *TNF* in the CAF/C group compared to the control (Figure 1B,C insert, respectively). The combined LPS and CAF treatment affected (*p* < 0.05) expression of IL-1β and TNFα on both mRNA and protein levels, which were lower (*p* < 0.05) in the LPS/CAF than in the LPS/C group (Figure 1A,C with inserts), but it did not change IL-6 on both mRNA and protein levels (Figure 1B with insert).

### 2.2. The Effect of Caffeine on Pro-Inflammatory Cytokines Receptors Gene Expression in the Ovine Hypothalamus under Basal and LPS-Challenge Conditions

The LPS injection significantly increased (*p* < 0.05) all pro-inflammatory cytokines receptors, except *IL6R*, in the hypothalamus, as indicated by their higher (*p* < 0.05) gene expression in the LPS/C group than in the control (Table 1). The single injection of CAF alone affected only *IL6ST*, which gene expression was higher (*p* < 0.05) in C/CAF than in the control group. In turn, CAF used together with LPS attenuated LPS action on *IL6ST* and *TNFRSF1A*, as indicated by their lower (*p* < 0.05) gene expression in LPS/CAF than in the LPS/C group.

### 2.3. The Effect of Caffeine on Gene Expression of Pro-Inflammatory Cytokines and Their Receptors in the Choroid Plexus under Basal and LPS-Challenge Conditions

As indicated in Table 2, mRNA expression of pro-inflammatory cytokines and their corresponding receptors, except *ILR6* and TNF in the ovine ChP, was significantly higher (*p* < 0.05) in the LPS/C group than in the control. The single injection of CAF alone increased (*p* < 0.05) only *IL6* and *IL6ST* gene expressions. In turn, CAF used together with LPS attenuated LPS action on *IL1R2* and *TNF*, as indicated by their lower (*p* < 0.05) gene expression in the LPS/CAF than in the LPS/C group.

## 3. Discussion

Our study has shown that CAF influences the synthesis of pro-inflammatory cytokines in the brain during the immune/inflammatory challenge induced by bacterial endotoxin injection in sheep during the follicular phase. As indicated previously [41], acute LPS treatment did not affect blood plasma progesterone and estradiol concentrations in the used female sheep model. It was found that the single injection of CAF in a dose of 30 mg/kg of bm. decreased mRNA expression of *IL1B* in the hypothalamus in LPS-treated female sheep, however, the reduced level of *IL1B* gene expression remained higher than in control animals. On the other hand, the administration of CAF completely abolished the endotoxin-stimulating effect on the level of IL-1β protein. Obtained results are in line with the recent study of Mallik et al. [42] where 14 days of CAF-treatment reduced LPS-stimulated IL-1β synthesis in the brain of male mice. Our results clearly indicate that the effect of CAF on the synthesis of IL-1β is not only connected with inhibiting *IL1B* gene expression but also influences the post-transcriptional stages. In general, LPS action in the brain is limited by BBB and most effects of peripherally administered LPS on the brain are therefore mediated by LPS receptors located outside the brain [43]. The LPS receptor, the toll-like receptor 4 (TLR4) is expressed in the endothelium of brain microvessels and the ChP [44,45]. In the case of IL-1β, LPS binding to TLR4 induces the synthesis of an inactive precursor, termed pro-IL-1β, which needs to be cleaved by caspase-1 to become an active molecule [46]. The activation of caspase-1 occurs via its recruitment to a multi-protein complex termed the inflammasome, which is composed of an adaptor molecule, a cytosolic pattern recognition receptor (NLRP3), and pro-caspase-1 [46]. It is evidenced that NLRP3 inflammasome expression in microglia, astrocytes, and brain microvessels endothelial cells which activation may create conditions necessary for active IL-1β synthesis [47,48,49]. The in vivo study on C57BL/6 mice reported that CAF may reduce NLRP3 inflammasome activation via the induction of autophagy in microglial cells [50]. Considering this, similar action of CAF on NLRP3 inflammasome in the ovine hypothalamus (including also brain microvessels) may be expected. In the ChP, no effect of CAF on LPS-induced *IL1B* expression was observed. This may be connected to the high cellular heterogeneity of ChP composed of the epithelial, endothelial, and stromal immune system cells, mainly bone marrow-derived macrophages (BMDMs) and dendritic cells [25]. It is worth mentioning that the immunomodulatory effect of CAF could be dependent upon the kind of target cells. According to Shimada and Hasegava-Ishii [51] ChP macrophages are cells that produce IL-1β in response to LPS stimulation, while other ChP cells rarely express IL-1β in early response to systemic LPS. During inflammatory conditions macrophages are stimulated and can be differentiated/polarized into classically activated (M1) macrophages, characterized by their ability to guide acute inflammatory responses and alternatively activated (M2) macrophages, which have inflammation resolving activity [52]. It is not known which subset of macrophages dominates in the ovine ChP during LPS-induced acute inflammation. Just recently, Ivan et al. [53] demonstrated that the BCSFB at the ChP could constitute a central nervous system (CNS) access gateway and a priming site for both M1 and M2 macrophages.

According to Kovács et al. [54], CAF enhanced the expression of IL-1β, in the subset of human M2 macrophages, whereas it did not influence the expression of IL1-β in the subset of M1 macrophages and mouse BMDM. Moreover, the same study [54] also showed that there were important differences in the time of cell response to CAF action. In M2 macrophages CAF enhanced IL-1β secretion as early as 6 h after LPS administration, while in monocytes only after 12 h [54]. Therefore, the differences in the response of the hypothalamus and ChP to CAF may be due to the different cellular compositions, locations, and nature of these structures. Considering IL-1β receptors, CAF attenuated LPS-induced *IL1R2* and has no effect on *IL1R1* gene expressions in the ovine ChP and both receptors in the hypothalamus. This is new and important information which, in light of the reports on the potential role of the IL-1 type II receptor (coded by *IL1R2* gene) in the transport of IL-1β throughout brain barriers [55], as well as the demonstration that activation of A1 and A2A receptors facilitates the entry of macromolecules throughout the BBB in mice [56], and the fact that antagonism of AR signaling blocks the entry of inflammatory cells and soluble factors into the brain [57], requires further research. Unfortunately, the main limitation of our study was the lack of antibodies suitable to detect this receptor in the ovine tissue.

In the LPS-challenge condition, a single injection of CAF abolished TNFα synthesis in the ovine hypothalamus which was observed at both mRNA and protein levels. Our results are consistent with a previous in vivo study on the LPS-injected mouse model, which showed that CAF attenuated the endotoxin-induced release of TNFα in the mouse brain [42]. According to our results, CAF down-regulated LPS-induced *TNF* expression also in the ovine ChP. Interestingly, opposite to IL-1β secretion, CAF abolished TNFα synthesis in both M1 and M2 macrophages as well as in monocytes [54]. Likewise, the in vitro study on LPS-stimulated human whole blood demonstrated that concentrations of CAF relevant to human consumption consistently decreased TNFα concentration in blood and that this effect was mediated by the cAMP/protein kinase A pathway [58]. Considering TNFα receptors, CAF attenuated only LPS-induced *TNFRSF1A* gene expression in the hypothalamus. It has been demonstrated that binding of TNFα to TNFR-1 (coded by *TNFRSF1A* gene) is sufficient to attain TNFα mediated cell killing while binding to TNFR-2 (coded by *TNFRSF1B*) is not [59].

Despite an in vitro study on RAW264.7 cells suggesting that CAF may have an inhibitory effect on IL-6 production under inflammatory conditions [60], and an in vivo study reporting the suppressory effect of CAF administration on IL-6 synthesis in mice brains [42], our study did not find an effect of acute CAF administration on the synthesis of IL-6 in the hypothalamus and *IL6* gene expression in the ChP of endotoxin-treated female sheep. We also did not observe CAF action on IL-6 receptors, *IL6R,* and *IL6ST* gene expression in both structures, except for weak attenuation of LPS-action on *IL6ST* in the hypothalamus. Our observations also show that the effect of CAF action on the synthesis of the pro-inflammatory cytokines in these structures seems to be dependent on animal health status and cytokines themselves. In healthy female sheep, CAF stimulated mostly IL-6 synthesis and gene expression of its signal-transducing component (*IL6ST*) in the hypothalamus and *IL6* and *IL6ST* gene expression in the ChP. The ability of CAF to modulate IL-6 synthesis in healthy subjects has been previously reported, however, the manner of this interaction is not clearly established. The studies on athletes showed that CAF supplementation increased the blood concentration of IL-6 in the response to exercise [61,62]. CAF action on IL-6 synthesis may be also dependent upon the target tissue or cell since in mice CAF was demonstrated to induce IL-6 synthesis by skeletal muscles and cultured myotubes but not by the liver, hepatocytes, adipocytes, and macrophages [63]. Determining the importance of the effects of CAF on the synthesis of IL-6 in the hypothalamus for the functioning of the central nervous system certainly requires further in-depth research, because this cytokine has been found to play both neurotoxic and neuroprotective roles in the brain [64]. Our study demonstrated that CAF administrated alone also reduced *TNF* gene expression but only in the hypothalamus.

Determining the exact mechanism by which CAF influences the expression of pro-inflammatory cytokines in the brain is difficult due to limited data concerning this issue. It seems that the anti-inflammatory effect of CAF on the production of cytokines is the result of a multidirectional effect. Previous studies showed that ADORs such as A2A, A2B, and A3 are involved in the regulation of the release of pro-inflammatory mediators such as TNF-α, IL-6, IL-12, nitric oxide, and macrophage inflammatory protein (MIP)-1α, and stimulated IL-10 secretion [65]. Particularly A3 receptor agonist was found to inhibit the synthesis of pro-inflammatory cytokines via down-regulation of NF-κB, a transcription factor that induces the production of a panel of pro-inflammatory cytokines including TNF-α, IL-1, and IL-6 [65]. However, the other study on LPS-activated cord blood monocytes suggested that A1, but not A3, acts as an operative in the effect of CAF on TNF-α [33]. Other research on University of Chile Bibulous rats showed that acting through the A1 and A2A CAF plays a neuroprotective role and may attenuate ethanol-induced inflammation in the cerebellum [66]. One of the endogenous mechanisms involved in the regulation of immune response and cytokine secretion is the cholinergic anti-inflammatory pathway in which the efferent vagus nerve regulates systemic cytokine levels through α7 nicotinic acetylcholine (ACh) receptor [67]. Our previous studies with different inhibitors of acetylcholinesterase (AChE), an enzyme responsible for ACh hydrolysis, showed suppression of IL-1β, IL-6, and TNFα synthesis in the ovine hypothalamus but not in the ChP which is linked with the lack of α7 nicotinic ACh receptor [68]. Considering the fact that CAF was demonstrated to act also as a non-competitive AChE inhibitor [69], a similar mechanism can be suggested for CAF action in the ovine hypothalamus.

## 4. Materials and Methods

### 4.1. Animals and Experimental Design

The experiment was performed on 2-years old Blackhead female sheep (*n* = 24) in the follicular phase of the estrous cycle during the breeding season (October). To standardize experimental conditions the stages of the estrous cycle were synchronized by the Chronogest^®^ CR (Merck Animal Health, Madison, NJ, USA) method. The experimental procedures were performed 24 h following PMSG injection. On the morning of the experiment, animals were placed in individual cages and randomly divided into four experimental groups (Table 3) receiving intravenously the following treatment: (1) control group (C/C)—double saline (0.9% *w*/*v* NaCl), (2) LPS group (LPS/C)—LPS (*Escherichia coli* 055:B5, Sigma Aldrich, Merck, Darmstadt, Germany) in a dose of 400 ng/kg bm. to induce immune stress and then saline, (3) CAF group (C/CAF)—saline and then CAF (Sigma Aldrich, Merck, Darmstadt, Germany) in a dose of 30 mg/kg bm. chosen based on [70] and the pilot experiment (Supplementary Figure S2), (4) LPS + CAF group (LPS/CAF)—LPS, and then CAF. During the experiment, the body temperature was measured and blood samples were collected through a catheter inserted into the jugular vein. The animals were sacrificed 3 h after treatment and after decapitation, the brains were rapidly dissected from the skulls. The ChP were removed from their anchoring to the Galien’s vein, and the split was made along the mid-line, separating the ChP from each lateral ventricle. The hypothalamus and ChP were immediately frozen in liquid nitrogen and stored at −80 °C until use.

### 4.2. Inflammatory Cytokines and Cortisol Concentration Assessment

The concentrations of pro-inflammatory cytokines in the hypothalamus were assayed using IL-1β ELISA kits (cat no. ESH0012; Wuhan Fine Biotech Co., Ltd., Wuhan, China), IL-6 ELISA kits (cat no. ESH0019; Wuhan Fine Biotech Co., Ltd., Wuhan, China) TNFα ELISA kits (cat no. ESH0025; Wuhan Fine Biotech Co., Ltd., Wuhan, China) designed and validated for sheep. The hypothalamic tissues were homogenized according to the method described elsewhere [68]. The assays procedures were performed according to the instructions provided by the manufacturer. Plates incubation and absorbance measurement at a wavelength of 450 nm were performed using the VersaMax reader (Molecular Devices LLC., Sunnyvale, CA, USA). The assay sensitivity was 18.75 pg/mL for IL-1β, 4.69 pg/mL for IL-6, and 9.375 pg/mL for TNFα. The concentration of pro-inflammatory cytokines in the hypothalamus was calculated relative to the total protein content of the sample assayed by the Bradford method. The mean concentration of total protein per well in the ELISA assays was 2.4 ± 0.1 mg/mL.

To validate the ewe’s responses to LPS administration, the plasma concentration of cortisol was assayed by RIA [71]. The sensitivity of the assay for cortisol was 0.95 ng/mL, and the intra- and inter-assay coefficients of variation were 10% and 12%, respectively.

### 4.3. Relative Gene Expression Determination

Relative gene expression assays were performed according to the previously published methods for hypothalamic [68] and the choroid plexus samples [72]. Frozen hypothalamic samples were homogenized by trituration in liquid nitrogen and lysed in lysing buffer (RA1) from the NucleoSpin^®^ RNA kit (MACHEREY-NAGEL GmbH and Co, Düren, Germany). Frozen ChP samples were homogenized using a FastPrep24 instrument (MP Biomedicals, Illkirch-Graffenstaden, France) and dedicated to Lysing Matrix D tubes (MP Biomedicals, Illkirch-Graffenstaden, France), filled with lysing buffer (RA1). Total RNA isolation from the hypothalamic and ChP samples was carried out using the NucleoSpin^®^ RNA kit (MACHEREY-NAGEL GmbH and Co, Düren, Germany), according to the manufacturer protocol. The concentration and purity of isolated RNA were quantified by measuring the optical density at 230, 260, and 280 nm in a NanoDrop 1000 apparatus (Thermo Fisher Scientific Inc., Waltham, MA, USA). The verification of RNA integrity was carried out by electrophoresis using 1.2 % agarose gel containing the GelRed Nucleic Acid Gel Stain (Biotium, Fremont, CA, USA). cDNA synthesis was performed using 1000 ng of total RNA and components of the Maxima™ First Strand cDNA Synthesis Kit for RT-qPCR (Thermo Fisher Scientific Inc., Waltham, MA, USA).

Real-Time RT-PCR analysis was carried out with the use of the HOT FIREPol EvaGreen^®^ qPCR Mix Plus (Solis BioDyne, Tartu, Estonia) for the hypothalamic samples and the DyNAmo SYBR Green qPCR kit with ROX (Thermo Fisher Scientific, Waltham, MA, USA) for the ChP samples, and HPLC-grade oligonucleotide primers (Genomed, Warszawa, Poland) in line with the method described elsewhere [68,72]. Specific primers to determine the expression of the tested and reference genes were chosen based on our previous experience (see Table 4). For hypothalamic samples, one reaction mixture of total volume amounting to 15 µL contained: 3 µL of PCR Master Mix, 10.05 μL of RNase-free water, 0.45 µL of primers (0.225 µL each primer) and 1.5 μL of the cDNA template. The real-time PCR reactions were carried out using a Rotor-Gene 6000 instrument (Qiagen, Dusseldorf, Germany) with the following protocol: 95 °C for 15 min and 30–35 cycles at 95 °C for 10 s (denaturation), 59 °C for 20 s (primer annealing) and 72 °C for 10 s (extension). The relative gene expression was calculated using the comparative quantification option [73] of a Rotor Gene 6000 software 1.7. (Qiagen, Dusseldorf, Germany) with reference to the mean expression of three housekeeping genes: glyceralde-hyde-3-phosphate dehydrogenase (*GAPDH*), β-actin (*ACTB*), and histone deacetylase1 (*HDAC1*). For ChP samples, each reaction mixture of total volume amounting to 10 µL contained: 5 µL of DyNAmo SYBR Green qPCR kit with ROX (Thermo Fisher Scientific, Waltham, MA, USA), 0.2 µM of each primer, and 3 µL cDNA template (1:10). The real-time PCR reactions were carried out using a Viia7 instrument (Applied Biosystems by Life Technologies, Waltham, MA, USA) and cDNA samples and reaction mix were transferred into 384-well plates, by the Bravo Automated Liquid Handling Platform (Agilent Technologies, Santa Clara, CA, USA) and the following protocol was used: 95 °C for 10 min and 37–40 cycles at 95 °C for 15 s (denaturation), 60 °C for 30 s (primer annealing) and 72 °C for 30 s (extension). The relative gene expression was calculated using the Real-Time PCR Miner (available online: http://ewindup.info/miner/, accessed on 27 March 2021) based on the Zhao and Fernald [74] algorithm, with reference to the mean expression of three housekeeping genes: *GAPDH*, *ACTB*, and *HDAC1*. A final melting curve analysis and agarose gel electrophoresis of PCR products were performed to verify the specificity of the amplification reaction after real-time PCR for hypothalamic and ChP samples.

### 4.4. Statistical Analysis

All data are presented as the mean ± SEM. Gene expression data were normalized to the average relative level of this mRNA expression in the control sheep, which was set to 1.0. The statistical analysis was performed using a STATISTICA 10 software (Stat Soft. Inc., Tulsa, OK, USA) for hypothalamic samples and a Graph Pad PRISM 8 (Graph Pad Software, San Diego, CA, USA) for ChP samples. The analyses were performed on raw data after verification of normality assumptions (Shapiro–Wilk’s test). Before analysis, data that failed the normality test were subjected to logarithmic transformation. The results of gene and protein expression were analyzed using one-way analyses of variances (ANOVA) and followed by a post-hoc Fisher’s least significance test comparing groups with each other. The results are presented as the mean ± SEM; statistical significance was set at *p* < 0.05.

## 5. Conclusions

Our study on the sheep model reveals that CAF may exert anti-inflammatory effects in the hypothalamus, among others by inhibiting the synthesis of pro-inflammatory cytokines such as IL-1β and TNFα. It was also determined that CAF may influence the gene expression of TNFα and some cytokines receptors in the ChP by which it may influence the inflammatory signal generated by the ChP. Due to the important role of the hypothalamus in the control of reproduction, a better understanding of the potential of CAF to suppress the neuroinflammatory processes may be important. However, further in-depth research is needed to better define and understand the mechanism by which CAF influences the synthesis of inflammatory mediators in the CNS.

## Figures and Tables

**Table 1:** The effect of caffeine on the relative gene expression (mean ± SEM; *n* = 6) of pro-inflammatory cytokines receptors in the ovine hypothalamus in basal and lipopolysaccharide-challenge conditions.

Gene	C/C	LPS/C	C/CAF	LPS/CAF
***IL1R1*** interleukin 1 receptor 1	1 ± 0.1 ^A^	2.8 ± 0.4 ^B^	1.3 ± 0.1 ^A^	2.3 ± 0.3 ^B^
***IL1R2*** interleukin 1 receptor 2	1 ± 0.1 ^A^	4.5 ± 0.7 ^B^	1.5 ± 0.1 ^A^	4.2 ± 0.6 ^B^
***IL6R*** interleukin 6 receptor	1 ± 0.1 ^A^	1 ± 0.1 ^A^	1.1 ± 0.1 ^A^	1 ± 0.1 ^A^
***IL6ST*** interleukin 6 signal transducer	1 ± 0.1 ^A^	1.4 ± 0.1 ^C^	1.2 ± 0.1 ^B^	1.3 ± 0.1 ^B^
***TNFRSF1A*** TNF receptor superfamily member 1A	1 ± 0.1 ^A^	1.6 ± 0.2 ^B^	0.8 ± 0.1 ^A^	1 ± 0.1 ^A^
***TNFRSF1B*** TNF receptor superfamily member 1B	1 ± 0.1 ^A^	1.9 ± 0.3 ^B^	1.1 ± 0.1 ^A^	1.8 ± 0.1 ^B^

C/C—double treated with saline (0.9% NaCl iv.), LPS/C—treated with lipopolysaccharide (LPS, 400 ng/kg of bm., iv.) followed by saline, C/CAF –treated with saline followed by caffeine (CAF, 30 mg/kg of body mass (bm.), iv.), LPS/CAF—treated with LPS followed by CAF. Gene expression data were normalized to the average relative level of this mRNA expression in the control sheep (C/C), which was set to 1.0. Different letters indicate significant differences at *p* < 0.05, according to one-way ANOVA followed by Fisher’s post hoc test comparing groups with each other.

**Table 2:** The effect of caffeine on the relative gene expression (mean ± SEM; *n* = 6) of pro-inflammatory cytokines and their receptors in the ovine choroid plexus in basal and lipopolysaccharide-challenge conditions.

Gene	C/C	LPS/C	C/CAF	LPS/CAF
***IL1B*** interleukin 1β; IL1R1	1 ± 0.1 ^A^	7.8 ± 2.1 ^B^	0.7 ± 0.1 ^A^	9.8 ± 3.1 ^B^
***IL1R1*** interleukin 1 receptor 1	1 ± 0.1 ^A^	3.6 ± 0.8 ^B^	2.3 ± 0.3 ^A^	4.1 ± 0.4 ^B^
***IL1R2*** interleukin 1 receptor 2	1 ± 0.2 ^A^	9.7 ± 1.6 ^C^	2.1 ± 0.3 ^A^	6.5 ± 0.3 ^B^
***IL-6*** interleukin 6	1 ± 0.1 ^A^	587.4 ± 146.0 ^C^	1.9 ± 0.2 ^B^	489.7 ± 58.0 ^C^
***IL6R*** interleukin 6 receptor	1 ± 0.1 ^AB^	0.8 ± 0.2 ^A^	1.4 ± 0.2 ^B^	0.8 ± 0.1 ^A^
***IL6ST*** interleukin 6 signal transducer	1 ± 0.1 ^A^	2.8 ± 0.4 ^C^	1.9 ± 0.2 ^B^	2.9 ± 0.2 ^C^
***TNF*** tumor necrosis factor	1 ± 0.1 ^AB^	2.1 ± 0.8 ^B^	0.7 ± 0.1 ^A^	0.8 ± 0.2 ^A^
***TNFRSF1A*** TNF receptor superfamily member 1A	1 ± 0.1 ^A^	1.5 ± 0.2 ^B^	1.1 ± 0.1 ^A^	1.4 ± 0.1 ^B^
***TNFRSF1B*** TNF receptor superfamily member 1B	1 ± 0.1 ^A^	3.8 ± 0.8 ^B^	1.7 ± 0.3 ^A^	4.0 ± 0.3 ^B^

C/C—double treated with saline (0.9% NaCl iv.), LPS/C—treated with lipopolysaccharide (LPS, 400 ng/kg of bm., iv.) followed by saline, C/CAF –treated with saline followed by caffeine (CAF, 30 mg/kg of body mass (bm.), iv.), LPS/CAF—treated with LPS followed by CAF. Gene expression data were normalized to the average relative level of this mRNA expression in the control sheep (C/C), which was set to 1.0. Different letters indicate significant differences at *p* < 0.05, according to one-way ANOVA followed by Fisher’s post hoc test comparing groups with each other.

**Table 3:** Experiment organization chart.

Group	No. of Animals	Experimental Treatment I	Dose [ng/kg]	Experimental Treatment II	Dose [mg/kg]
**C/C**	6	NaCl	0	NaCl	0
**LPS/C**	6	LPS	400	NaCl	0
**C/CAF**	6	NaCl	0	caffeine	30
**LPS/CAF**	6	LPS	400	caffeine	30

C/C—saline-treated, control; LPS/C—lipopolysaccharide-treated; C/CAF—caffeine-treated; LPS/CAF—lipopolysaccharide- and caffeine-treated.

**Table 4:** Sequences of oligonucleotide primers used for real-time PCR in the hypothalamus and choroid plexus.

	Gene	(Forward/Reverse) Sequence 5′ → 3′	Amplicon Size (bp)	References/ Sources
*Genes under study*	*IL1B*	F: CAGCCGTGCAGTCAGTAAAA R: GAAGCTCATGCAGAACACCA	137	[24,68]
	*IL1R1*	F: GGGAAGGGTCCACCTGTAAC R: ACAATGCTTTCCCCAACGTA	124	[24,68]
	*IL1R2*	F: CGCCAGGCATACTCAGAAA R: GAGAACGTGGCAGCTTCTTT	162	[24,68]
	*IL6*	F: GTTCAATCAGGCGATTTGCT R: CCTGCGATCTTTTCCTTCAG	165	[24,68]
	*IL6R*	F: TCAGCGACTCCGGAAACTAT R: CCGAGGACTCCACTCACAAT	149	[24,68]
	*IL6ST*	F: GGCTTGCCTCCTGAAAAACC R: ACTTCTCTGTTGCCCACTCAG	139	[24,68]
	*TNF*	F: CAAATAACAAGCCGGTAGCC R: AGATGAGGTAAAGCCCGTCA	153	[24,68]
	*TNFRSF1A*	F: AGGTGCCGGGATGAAATGTT R: CAGAGGCTGCAGTTCAGACA	137	[24,68]
	*TNFSFR2A*	F: ACCTTCTTCCTCCTCCCAAA R: AGAAGCAGACCCAATGCTGT	122	[24,68]
*Reference* *genes*	*GAPDH*	F: AGAAGGCTGGGGCTCACT R: GGCATTGCTGACAATCTTGA	134	[68] hypothalamus
	*GAPDH*	F: TGACCCCTTCATTGACCTTC R: GATCTCGCTCCTGGAAGATG	143	[72] ChP
	*ACTB*	F: CTTCCTTCCTGGGCATGG R: GGGCAGTGATCTCTTTCTGC	168	[68] hypothalamus
	*ACTB*	F: GCCAACCGTGAGAAGATGAC R: TCCATCACGATGCCAGTG	122	[72] ChP
	*HDAC1*	F: CTGGGGACCTACGGGATATT R: GACATGACCGGCTTGAAAAT	115	[24,68]

*IL1B*—interleukin 1-beta; *IL1R1*—interleukin 1 receptor, type I; *IL1R2*—interleukin 1 receptor, type II; *IL6*—interleukin 6; *IL6R*—interleukin 6 receptor; *IL6ST*—glycoprotein 130; *TNF*—tumor necrosis factor; *TNFRSF1A*—tumor necrosis factor receptor superfamily member 1A; *TNFRSF1B*—tumor necrosis factor receptor superfamily member 1B; *GAPDH*—glyceraldehyde-3-phosphate dehydrogenase; *ACTB*—beta-actin; *HDAC1*—histone deacetylase 1; ChP—choroid plexus.

## Data Availability

The data presented in this study are available on request from the corresponding authors.

## Supplementary Materials

The following are available online at https://www.mdpi.com/article/10.3390/ijms222413237/s1.
