# Supplementary material for: Acute Effect of Caffeine on the Synthesis of Pro-Inflammatory Cytokines in the Hypothalamus and Choroid Plexus during Endotoxin-Induced Inflammation in a Female Sheep Model
Source: Int J Mol Sci. 2021 Dec 8;22(24):13237. doi: 10.3390/ijms222413237 (PMC8706723; doi:10.3390/ijms222413237)
Supplement: Supplementary file 1 [file ijms-22-13237-s001.zip › ijms-1490979-supp-proof done.pdf]

Acute effect of caffeine on the synthesis of pro-inflammatory cytokines in the hypothalamus and choroid plexus during endotoxin-induced inflammation in a female sheep model

Aleksandra Szczepkowska, Maciej Wójcik, Dorota Tomaszewska-Zaremba, Hanna Antushevich, Agata Krawczyńska, Wiktoria Wiechetek, Janina Skipor and Andrzej P. Herman

Supplementary Materials

Supplementary Figure S1: Body temperature and blood plasma cortisol concentration.

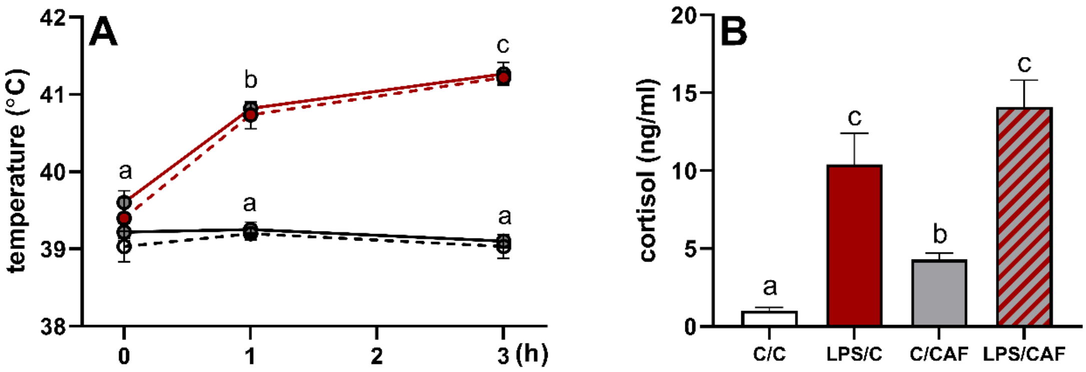

Figure S1. Mean (±SEM) body temperature (**A**) in saline- (black solid line), lipopolysaccharide- (LPS, 400 ng/kg iv., red solid line), caffeine-(CAF, 30 mg/kg bm., iv., black dotted line) and LPS/CAF- (red dotted line) treated animals. (**B**) Mean (±SEM) blood plasma cortisol concentration in saline (C/C, white bars), LPS (LPS/C, red bars), CAF (CAF/C, grey bars) and LPS/CAF (red-grey hatched bars) treated animals. Different letters indicate significant differences at $p < 0.05$, according to a one -way ANOVA followed by Fisher's post hoc test comparing groups with each other.

Supplementary Figure S2: Pilot experiment.

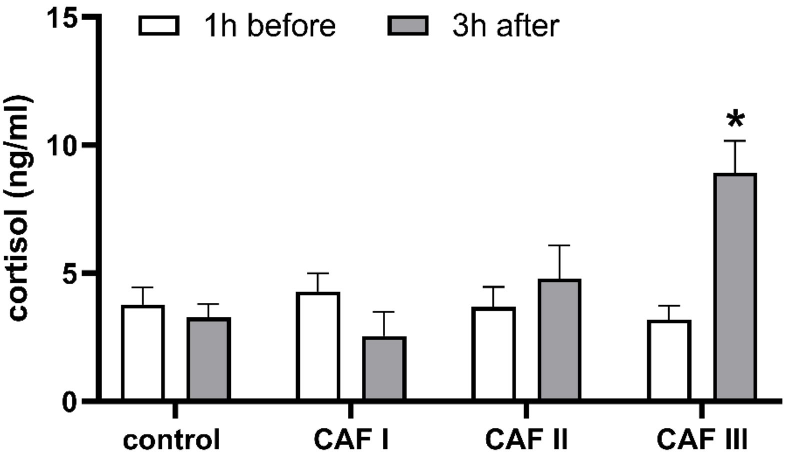

Figure S2. Mean (±SEM) blood plasma cortisol concentration in female sheep in the follicular phase of the estrous cycle treated with saline (control, *n*=6) or caffeine at doses of 10 mg/kg bm. (CAF I, *n*=6), 20 mg/kg bm. (CAF II, *n*=6) and 30 mg/kg bm. (CAF III, *n*=6). All experiments consisted of a baseline period when no treatment was given (1.5 to 0.5 h before; white bars) and a period after the treatment (2 to 3 h after; grey bars). * indicates significant differences at $p < 0.05$, according to two-way ANOVA followed by Fisher's post hoc test comparing groups with each other.
